# Longitudinal bidirectional relations between body dissatisfaction and depressive symptoms among Black adolescents: A cross-lagged panel analysis

**DOI:** 10.1371/journal.pone.0228585

**Published:** 2020-01-30

**Authors:** Yan Wang, Sarah D. Lynne, Dawn Witherspoon, Maureen M. Black

**Affiliations:** 1 Department of Pediatrics, University of Maryland School of Medicine, Baltimore, Maryland, United States of America; 2 Department of Epidemiology and Public Health, University of Maryland School of Medicine, Baltimore, MD, United States of America; 3 Department of Family, Youth and Community Sciences, University of Florida, Gainesville, Florida, United States of America; 4 Department of Psychology, University of North Florida, Jacksonville, Florida, United States of America; 5 RTI International, Research Triangle Park, North Carolina, United States of America; University of St Andrews, UNITED KINGDOM

## Abstract

**Objective:**

To assess the variation in body dissatisfaction and depressive symptoms by weight status and the bi-directional relations between body dissatisfaction and depressive symptoms by weight status among Black adolescents.

**Methods:**

A sample of 153 Black adolescents aged 12–13 years, either overweight/obese (n = 57, 37%) or healthy weight (n = 96, 63%), were recruited and evaluated three times over two years (T1, T2 and T3). Measured weight and height were converted to age and sex-specific BMI z-score; body dissatisfaction was measured with silhouettes, and depressive symptoms were measured with the Beck Depression Inventory (BDI-I). Bidirectional relations were assessed with cross-lagged panel analyses, accounting for stability over time and contemporary correlations.

**Results:**

Body dissatisfaction was higher among the overweight/obese group than the healthy weight group. No significant differences were found for depressive symptoms by weight status. Among the overweight/obese group, there were bidirectional relations: antecedent body dissatisfaction predicted subsequent depressive symptoms (T1-T2: β = 0.42, SE = 0.11, p<0.001; T2-T3: β = 0.36, SE = 0.09, p<0.001) and antecedent depressive symptoms predicted subsequent body dissatisfaction (T1-T2: β = 0.25, SE = 0.10, p = 0.012; T2-T3: β = 0.17, SE = 0.08, p = 0.045). Among the healthy weight group, there was no relation in either direction.

**Conclusions:**

Elevated body dissatisfaction among the overweight/obese group supports weight-based stigma as a stressor among Black adolescents. The bidirectional relations between body dissatisfaction and depressive symptoms among the overweight/obese group support the internalization of thinness idea and negative self-appraisal associated with depressive symptoms. Prevention of both body dissatisfaction and depressive symptoms may be mutually beneficial among Black adolescents with overweight/obesity.

## Introduction

Overweight/obesity is a serious health problem among adolescents. A national survey reported that black adolescents have a higher prevalence of overweight/obesity than white adolescents (overweight or obesity, 37.1% vs. 29.5%; obesity, 18.8% vs. 15.7% [1]). Adolescents from low-income families are at increased risk for overweight or obesity [2, 3]. Behavioral, physiological, and emotional mechanisms are thought to contribute to overweight/obesity among adolescents through a process described in the Cyclic Obesity/Weight-Based Stigma model (COBWEBS [4]). For example, overweight/obesity may lead to weight stigma, a stressor that initiates emotional (e.g. body dissatisfaction or depressive symptoms), behavioral (e.g. overeating), and physiological responses (e.g. elevated secretion of cortisol), resulting in further weight gain [4]. Body image dissatisfaction is the discrepancy between perception of body weight and shape and perception of ideal appearance [5]. Both body dissatisfaction and depressive symptoms have serious health consequences and are prevalent among adolescents. Body dissatisfaction has been associated with eating disorders e.g. bulimia and anorexia [6], and depressive symptoms have been associated with academic problems and failure, substance abuse, self-harming behaviors and suicide [7] and depression in adulthood [8]. Approximately 40% of adolescent females and 23% of adolescent males are dissatisfied with their bodies [9]; and the prevalence of major depressive episodes within the past year is 20.0% among adolescent females and 6.8% among adolescent males [10]. The prevalence of both health problems is elevated among overweight/obese adolescents [11, 12]. The adverse health consequences warrant examining the risk factors of body dissatisfaction and depressive symptoms among adolescents, especially adolescents with overweight/obesity.

The association between body dissatisfaction and depressive symptoms has been well established in cross-sectional studies among adolescents [13–16]. Based on the thin-ideal internalization model [17], body dissatisfaction leads to depressive symptoms. Adolescents accept thinness as socially defined ideals of attractiveness in the media[18] and internalize these ideals with positive attributes such as desirability and attractiveness [19–21]. A discrepancy between ideal weight or shape and actual weight or shape can result in body dissatisfaction, which leads to depressive symptoms [20]. This theory has been supported by longitudinal studies that reported body dissatisfaction as a risk factor for subsequent depressive symptoms [22–27]. However, a reverse relation has also been proposed. Depressive symptoms may lead to negative self-appraisal and when it was reflected on body weight, result in body dissatisfaction [28, 29].

Assessment of the bidirectional relations between body dissatisfaction and depressive symptoms (testing both directions) in prospective studies is important for causal inference. However, studies have seldom assessed bidirectional relations. To our knowledge, only two studies have examined the bidirectional relations in adolescence [30, 31]. One study assessed two cohorts of racially mixed adolescents aged 12.9 years and 15.9 years respectively and reassessed them at the 5-year and 10-year follow up [31]. The other study assessed a sample of two cohorts of mainly white adolescents in England aged 8–9 years and aged 11–12 years, separately, over three years [30]. In addition, two other studies examined the relation between antecedent body dissatisfaction and later depressive symptoms [26] and the reverse relation [32], respectively, using the same sample of mainly white children and adolescents followed for 5 years. These studies reported mixed findings, suggesting that the bidirectional relations between body dissatisfaction and depressive symptoms are complex, and depend on developmental period (age) and sex. We did not identify any studies that assessed bidirectional relations among Black adolescents, especially from low-income families.

The prevalence of overweight/obesity is higher among Black adolescents, compared to White adolescents [1]. Adolescents from low-income families are at higher risk of overweight/obesity, possibly because of the lack of resources for physical activity or healthy diet in their family or neighborhood [2, 3]. In addition, there have been reports that Black adolescents prefer a bigger body size than White adolescents [33, 34]. Relations between body dissatisfaction and depressive symptoms found mainly among White adolescents [9, 22, 25, 26, 30] need to be examined among Black adolescents from low-income families [33, 35]. In addition, weight status is related to both body dissatisfaction and depressive symptoms, and is an important factor to consider in the examination of relations between body dissatisfaction and depressive symptoms [36, 37]. We did not identify any bidirectional studies that assessed the relations by weight status.

Sex is related to body dissatisfaction and depressive symptoms. The relationship between the internalization of the thinness ideal and body dissatisfaction has been examined more frequently in adolescent females than in adolescent males. Some adolescent males desire muscularity, rather than thinness [38]. Sex differences in depressive symptoms emerge in early adolescence with females reporting more symptoms than males. In addition, studies examining bidirectional relations between body dissatisfaction and depressive symptoms reported sex variation in the relations [30, 31]. Weight status also varies by sex; adolescents girls have a higher prevalence of overweight/obesity than adolescent boys from low-income families, based on a national study [3]. The addition of variation by both weight status and sex in the investigation of bidirectional relations between body dissatisfaction and depressive symptoms among Black adolescents is novel.

To fill the gaps, this prospective study investigates the bidirectional relations between body dissatisfaction and depressive symptoms among Black adolescents from low-income families. Adolescents with overweight/obesity and adolescents of healthy weight were assessed separately. Based on the COBWEBS model and prior investigations, we tested two hypotheses: (1) body dissatisfaction and depressive symptoms are higher among the overweight/obese group, compared to the healthy weight group, (2) there are bidirectional relations between body dissatisfaction and depressive symptoms in the overweight/obese group, not in the healthy weight group. In addition, we conducted exploratory analyses on the sex variation in the relations for each weight status.

## Materials and methods

### Participants

Two hundred and thirty-five adolescents between 11–16 years of age from low-income communities were recruited from primary care clinics at an urban, university medical center, and from urban middle schools to participate in a randomized controlled trial of health promotion/obesity prevention intervention [39]. Data on socio-demographic characteristics, body dissatisfaction and depressive symptoms were collected through computerized audio computer-assisted self-interview (ACASI) at baseline (T1, 7/1/2002-4/16/2004), ~10 months (T2, 12/2/2002-11/4/2005) and ~24 months after enrollment (T3, 2/3/2004-11/7/2006). The University of Maryland Baltimore Institutional Review Board approved this research; all parents signed informed consent, and all adolescents signed informed assent.

Since the wide age range of the participants may distort the relations, we restricted the analyses to adolescents age 12–13 years at baseline (n = 157, 68.9%). 10.19% (n = 16) of the adolescents were overweight (BMI-for-age percentile: ≥85^th^ to <95^th^), 26.11% (n = 41) obese (≥95^th^ percentile), 61.15% (n = 96) of healthy weight (≥5^th^ to <85^th^ percentile) and 2.55% (n = 4) underweight (<5^th^ percentile). Given the small sample of adolescents who were underweight, we further restricted the analytic sample to 153 adolescents who were either overweight/obese (n = 57) or healthy weight (n = 96).

### Measures

#### Adolescent weight status at baseline

Trained research assistants measured the adolescents’ height to the nearest 0.1cm (Shorr Productions, Olney, Maryland) and weight to the nearest 0.1kg (TANITA 300GS; Tanita Corp, Arlington Heights, IL) three times. The average of the three measurement was calculated and converted to age and sex-specific BMI percentiles based on CDC growth charts [40] to determine weight status.

#### Depressive symptoms (DS)

Depressive symptoms were measured, rather than clinical depression. Although the current study addresses depressive symptoms, adolescents with subclinical forms of depression may experience negative consequences similar to those of clinical depression. Depressive symptoms were measured with the Beck Depression Inventory (BDI [41]), which consists of 21 items that address depressive symptoms in the past week, e.g., sadness, feeling guilty, and fatigue, using a 4-point response scale of severity. A summary score was calculated with higher scores indicating more depressive symptoms. The BDI has satisfactory concurrent and discriminant validity among adolescents [42]. For the current study, Cronbach’s alpha was 0.88 and scores ranged from 0 to 44.

#### Body dissatisfaction (BD)

Body dissatisfaction was measured by comparing perceived body image with ideal image, using a culturally adapted, age- and sex-specific, 9-point silhouette scale, modified from Stunkard, Sørensen, & Schulsinger, (1983) [43] to resemble Black youth [44]. The silhouettes were ordered from thinnest (rating = 1) to heaviest (rating = 9). The adolescents identified the silhouette closest to their current body size and then on a separate, but identical scale, the silhouette they desired. The discrepancy score was defined as perceived body size minus desired body size, with a positive discrepancy score indicating a desire to be thinner and a negative score indicating a desire to be heavier. Good test-retest reliability and high construct validity have been reported for this sample [44].

#### Other variables

All the following variables were collected at T1.

**Adolescent age:** Age (years) was calculated by estimating the difference between self-reported date of birth and the interview date.

**Adolescent sex:** Adolescents’ self-reported sex was categorized into males and females.

**Caregiver education:** Caregivers’ self-reported education level was categorized into “lower than high school” and “high school diploma, GED or higher.”

**Poverty:** Poverty ratio was calculated with caregiver-reported total family income, family size, and number of children, based on the 2003 federal poverty index [45] to match the time of data collection. It was further categorized as “at or below federal poverty threshold” and “above federal poverty threshold.”

**Caregiver overweight/obesity:** Caregivers self-reported their own height and weight. BMI was calculated and was categorized into “overweight/obesity” (BMI> = 25) vs. “healthy weight” (BMI<25).

**Intervention:** Intervention status (intervention vs. control) was dummy coded based on the intervention assignment. The intervention group received a 12-session intervention based on socio-cognitive theory, delivered by Black college mentors, to promote healthy eating and physical activity. The control group did not receive any intervention.

### Statistical analyses

The investigations in this study were not planned in the original protocol of the obesity prevention trial (ClinicalTrials.gov identifier: NCT00746083). The protocol for this study can be found at protocols.io (dx.doi.org/10.17504/protocols.io.8nnhvde). Sample characteristics and body dissatisfaction/depressive symptoms were compared by weight status at each time point, with T-tests for continuous variables and Chi-square tests or Fisher exact tests (when at least one cell size<10) for categorical variables, whichever was appropriate.

For each weight group, we conducted a trend test, the nonparametric test for trend across ordered groups, as an extension of the Wilcoxon rank-sum test [46], and tested whether body dissatisfaction or depressive symptoms had an increasing or decreasing trend over time [47].

To assess the relations between body dissatisfaction and depressive symptoms, we first assessed Pearson correlation coefficients among body dissatisfaction and depressive symptoms at T1-T3 and covariates at T1. Then we conducted cross-lagged panel models (CLPM) in structural equation modeling (SEM) framework to examine the bidirectional relations between body dissatisfaction and depressive symptoms, accounting for the stability of depressive symptoms or body dissatisfaction across time [48]. CLPM were tested with Mplus 8.0 [49] for each weight group. In CLPM, variance in each outcome (e.g. depressive symptoms) at T2 or T3 was derived from two main sources: the effect of the same variable (e.g. depressive symptoms) at an antecedent time point (T1 or T2, autoregressive paths), and the effect of the other variable (e.g. body dissatisfaction) at an antecedent time point (T1 or T2, cross-lagged paths). Correlations between variables measured concurrently (e.g., T1 depressive symptoms with T1 body dissatisfaction) were also assessed. CLPM has advantages over traditional growth modeling in that it can analyze the entire theoretical model (bidirectional relations, stability and correlation) in one analysis while the traditional growth modeling typically involves testing only one specific relation (e.g. depressive symptoms predicting later body dissatisfaction, or body dissatisfaction predicting later depressive symptoms). In addition, CPLM can test both the specific hypothesized relationships and the plausibility of the overall model (i.e., the fit of the model). Chi-square test (X^2^), comparative fit index (CFI), Root Mean Square Error of Approximation (RMSEA) were used to test how the models fit the data. Non-significant X^2^ test (p>0.05), RMSEA< = 0.08, TLI>0.95 and CFI>0.95 indicate good model fit [50]. The significance level was defined as p<0.05.

Since depressive symptoms were slightly skewed (skewness 1.6–2.7), parameters were estimated with maximum likelihood estimation with robust standard errors (MLR). To explore whether sex moderates the relations, multiple-group CLPMs were conducted, comparing the cross-lagged paths, and autoregressive paths by sex, with Wald Chi-square (X^2^) tests. Since the data were collected from an obesity prevention study, we assessed the moderating effect of intervention on the bidirectional relations with multiple-group CLPM. In the absence of moderation by intervention, we combined the intervention and control groups and included intervention status as a covariate in all the analyses.

#### Handling of missing values

For the overweight/obese group, 25% (T2) and 24% (T3) were lost to follow-up. For adolescents of healthy weight, 18% (T2) and 25% (T3) were lost to follow-up. No significant differences were found in the sample characteristics, body dissatisfaction or depressive symptoms at baseline by attrition for either weight group. Missing data were accounted using full information maximum likelihood (FIML).

## Results

### Sample characteristics

The mean age of the adolescents at T1 was 13.02 years (SD = 0.57); 53% were females and 47% were males; half (53%) were randomized to the intervention group. About three-quarters (78%) of the mothers had completed high school and slightly over half (58%) were living below the federal poverty level. About one-third (37%) of the adolescents were overweight or obese and 63% were of healthy weight. The overweight/obese group had a marginally higher percentage of females than the healthy weight group (63% vs. 47%, p = 0.051), with no differences in other characteristics including adolescent age, caregiver education or overweight/obesity, poverty, or intervention status (Table 1).

**Table 1:** Selected sample characteristics, depressive symptoms and body dissatisfaction for the healthy weight group (n = 96) and the overweight/obese group (n = 57), separately.

	Total (n = 153)	Healthy weight (n = 96)	Overweight/obese (n = 57)	
Characteristics	Mean (SD) /n (%)	Mean (SD) /n (%)	Mean (SD) /n (%)	p
***Adolescents***				
**Age (Mean, SD)**	13.02(0.57)	13.05(0.60)	12.95(0.52)	0.261
**Sex**				
Female	81(53)	45(47)	36(63)	
Male	72(47)	51(53)	21(37)	0.051
**Intervention**				
Control	72(47)	50(52)	22(39)	
Intervention	81(53)	46(48)	35(61)	0.106
***Caregivers***				
** Caregiver education**				
<high school	37(24)	20(21)	17(30)	
> = high school	116(78)	76(79)	40(70)	0.209
**Poverty**				
No	59(42)	33(38)	26(50)	
Yes	80(58)	54(62)	26(50)	0.164
**Caregiver overweight/obesity (BMI> = 25)**				
No	34(22)	25(26)	9(16)	
Yes	118(78)	70(74)	48(84)	0.161
**Adolescent body dissatisfaction**				
Time 1	0.55(1.94)	-0.44(1.54)	2.22(1.32)	<0.001
Time 2	0.44(1.70)	-0.36(1.30)	2.00(1.24)	<0.001
Time 3	0.35(1.87)	-0.51(1.51)	1.85(1.46)	<0.001
**Adolescent depressive symptoms**				
Time 1	6.93(7.87)	6.28(7.47)	8.04(8.46)	0.184
Time 2	4.47(6.84)	4.19(6.96)	4.95(6.65)	0.562
Time 3	4.33(6.40)	4.17(6.31)	4.60(6.63)	0.736

^a^P values are based on T-tests for continuous variables or Chi-square tests/Fisher Exact tests for the categorical variables, whichever is appropriate.

### Body dissatisfaction by weight status

Table 1 shows that body dissatisfaction score was significantly higher by ~2 silhouettes in the overweight/obese group than the healthy weight group at each time point of T1-T3 (ps<0.001).

Trend tests showed that body dissatisfaction (continuous variable) was stable with no significant trends across time for each group (Z = -1.45 for the healthy weight group and Z = -1.74 for the overweight/obese group, ps>0.05).

### Depressive symptoms by weight status

Table 1 shows that there was no difference in the depressive symptoms score by weight status at each time point of T1-T3 (ps>0.10). Trend tests showed that depressive symptoms significantly decreased across time for both the overweight/obese group (mean 8.04 at T1, 4.95 at T2 and 4.60 at T3, Z = -2.60, p = 0.009) and the healthy weight group (mean 6.28 at T1, 4.19 at T2 and 4.17 at T3, Z = -2.28, p = 0.023).

### Relations between body dissatisfaction and depressive symptoms

#### Overweight/obese group

As shown in Table 2, there were significantly positive correlations between adjacent time points for depressive symptoms (T1 and T2, T2 and T3), and between any two time points for body dissatisfaction (T1 and T2, T1 and T3, T2 and T3). In addition, depressive symptoms at T1 were correlated with body dissatisfaction at T2, and depressive symptoms at T2 and T3 were correlated with body dissatisfaction at T2 and T3, separately. Covariates with at least one significant or marginally significant correlation (ps<0.10) with body dissatisfaction or depressive symptoms (poverty, maternal education, and intervention) were included in the CLPM.

**Table 2:** Correlation coefficients of sample characteristics and body dissatisfaction and depressive symptoms over time.

**Overweight/obese group (n = 57)**												
	**1**	**2**	**3**	**4**	**5**	**6**	**7**	**8**	**9**	**10**	**11**	**12**
**1: Age**	1.00											
**2: Male**	0.11	1.00										
**3: Poverty**	0.17	0.16	1.00									
**4: Overweight/obesity**	0.08	-0.17	0.11	1.00								
**5: Maternal education (> = high school)**	-0.17	-0.14	-0.09	0.24^a^	1.00							
**6: Intervention**	0.07	0.08	0.00	-0.05	0.27^b^	1.00						
**7: DEP at T1**	0.21	0.04	0.14	0.12	0.11	-0.07	1.00					
**8: DEP at T2**	0.07	0.02	0.17	0.09	0.18	-0.07	0.37^b^	1.00				
**9: DEP at T3**	0.00	-0.27	-0.10	-0.16	0.25	-0.17	0.21	0.58^b^	1.00			
**10: DIS at T1**	-0.14	-0.16	-0.24^a^	0.07	0.29	-0.15	0.29	0.48	0.34	1.00		
**11: DIS at T2**	-0.09	-0.25	-0.21	0.11	0.40^b^	-0.08	0.34^b^	0.40^b^	0.45^b^	0.54^b^	1.00	
**12: DIS at T3**	0.02	-0.24	-0.19	0.17	0.08	-0.38^b^	0.24	0.45^b^	0.50^b^	0.43^b^	0.69^b^	1.00
**Healthy weight group (n = 96)**												
	**1**	**2**	**3**	**4**	**5**	**6**	**7**	**8**	**9**	**10**	**11**	**12**
**1: Age**	1.00											
**2: Male**	0.17	1.00										
**3: Poverty**	-0.10	-0.03	1.00									
**4: Overweight/obesity**	0.17^a^	0.20	-0.08	1.00								
**5: Maternal education (> = high school)**	0.05	-0.12	-0.22^b^	-0.07	1.00							
**6: Intervention**	0.00	0.02	0.20^a^	-0.01	-0.07	1.00						
**7: DEP at T1**	0.17^a^	-0.06	-0.12	-0.11	0.04	-0.08	1.00					
**8: DEP at T2**	0.11	-0.11	-0.16	-0.16	-0.10	0.09	0.56	1.00				
**9: DEP at T3**	0.10	-0.10	-0.17	-0.04	0.05	0.13	0.48^b^	0.39^b^	1.00			
**10: DIS at T1**	-0.03	0.22^b^	0.11	0.01	-0.13	-0.09	-0.11	-0.10	-0.17	1.00		
**11: DIS at T2**	-0.02	0.01	0.15	0.04	0.03	-0.04	0.05	0.21^a^	0.00	0.46^b^	1.00	
**12: DIS at T3**	-0.08	-0.12	-0.05	-0.09	0.18	-0.27^b^	0.24^b^	0.05	0.28^b^	0.03	0.46^b^	1.00

DEP: depressive symptoms, DIS: body dissatisfaction.

^a^ indicates p = 0.05–0.10

^b^ indicates p<0.05.

**Fig 1:**
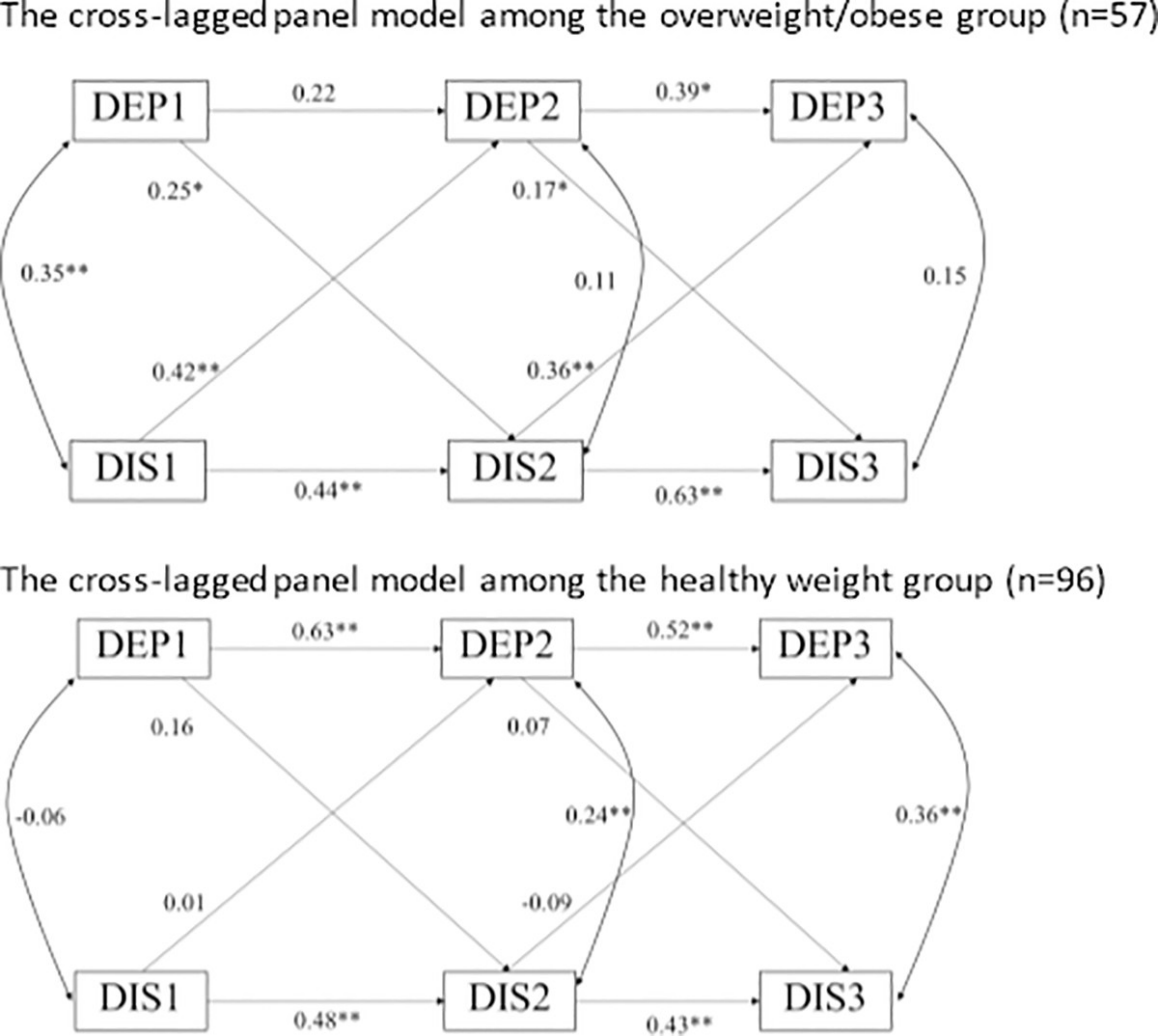
Cross-lagged panel analyses on body dissatisfaction and depressive symptoms over time among Black adolescents of overweight/obese group (upper part) and healthy weight group (lower part), separately. DEP1-DEP3 indicate depressive symptoms at time 1, time 2 and time 3, separately. DIS1-DIS3 indicate body dissatisfaction at time 1, time 2 and time 3, separately. The model for the overweight/obese group controls for maternal education, intervention and poverty. The model for the healthy weight group controls for sex, intervention and adolescents’ age. To simply the figures, the covariates were not depicted in the figure. Standardized estimate βs were presented. * indicates p = 0.05–0.10. ** indicates p <0.05.

We conducted the CLPM with autoregressive paths and cross-lagged paths freely estimated among the overweight/obese group. The covariates were included by regressing the outcomes (body dissatisfaction and depressive symptoms) at each time point on the covariates. As suggested by Little [51], we deleted the relations with ps>0.1 between covariates and the outcomes, leaving the regression of body dissatisfaction at T1 and T2 on maternal education, at T3 on intervention, and depressive symptoms at T2 on poverty (ps<0.1) in the model. There was no significant difference between the T1-T2 and T2-T3 time intervals (0.99 vs. 1.07 years, p = 0.256 based on T test). Therefore, the equality in the cross-lagged paths over time was tested, comparing the cross-lagged relations in T1-T2 to the cross-lagged relations in T2-T3. The Wald test (X^2^ = 1.869, df = 2, p = 0.393) suggested that equality over time in the cross-lagged relations could not be rejected for either antecedent body dissatisfaction and later depressive symptoms, or antecedent depressive symptoms and later body dissatisfaction. The final model with equality constrained for the cross-lagged relations is illustrated in Fig 1. The regression coefficients and the model fitness criteria can be found in Table 3. The model had a satisfactory fit to the data.

**Table 3:** The regression coefficients in the CLPM by weight status among Black adolescents age 12–13 years.

	T1-T2		T2-T3	
	β (SE)	p	β (SE)	p
**Overweight/obese group (n = 57)**				
**Cross-lagged relations**				
DIS predicting later DEP	0.42(0.11)	<0.001	0.36(0.09)	<0.001
DEP predicting later DIS	0.25(0.10)	0.012	0.17(0.08)	0.045
**Stability**				
DIS predicting later DIS	0.44(0.12)	<0.001	0.63(0.08)	<0.001
DEP predicting later DEP	0.22(0.14)	0.12	0.39(0.15)	0.011
Model fitness: Chi-square X^2^ = 18.058, df = 19, p = 0.519, RMSEA<0.001, CFI = 1.0, TLI = 1.0				
**Healthy weight group (n = 96) **				
**Cross-lagged relations**				
DIS predicting later DEP	0.01(0.07)	0.878	-0.09(0.19)	0.633
DEP predicting later DIS	0.16(0.10)	0.119	0.07(0.16)	0.685
**Stability**				
DIS predicting later DIS	0.48(0.09)	<0.001	0.43(0.15)	0.004
DEP predicting later DEP	0.63(0.13)	<0.001	0.52(0.15)	<0.001
Model fitness: Chi-square X^2^ = 18.935, df = 19, p = 0.461, RMSEA<0.001, CFI = 1.0				

DIS indicates body dissatisfaction, and DEP indicates depressive symptoms. Standardized regression coefficient βs were reported. a. The model for overweight/obese group included maternal education, poverty, intervention as covariates. b. The model for healthy weight group included sex, intervention and adolescents’ age as covariates.

The cross-lagged paths were significant in both directions among adolescents with overweight/obesity (Table 3): (1) antecedent body dissatisfaction predicted later depressive symptoms and (2) antecedent depressive symptoms predicted later body dissatisfaction. The relation between antecedent body dissatisfaction and later depressive symptoms was stronger than the relation between antecedent depressive symptoms and later body dissatisfaction. In addition, there was moderate stability for body dissatisfaction (β = 0.44–0.63). The stability for depressive symptoms was weaker, compared to body dissatisfaction (Table 3).

#### The moderating effect of sex among overweight/obese group

There were no differences in body dissatisfaction by sex. Females had marginally higher depressive symptoms than males at T3 (mean 6.00 vs. 2.31, p = 0.080). Multiple-group CLPM was conducted by sex. Given the small sample size for the overweight/obese group, no covariates were included. No significant differences by sex were found for the cross-lagged paths (Wald test X^2^ = 0.379, df = 2, p = 0.827), or the autoregressive paths (Wald test X^2^ = 6.60, df = 4, p = 0.159).

#### Healthy weight group

As shown in Table 2, there were positive correlations between body dissatisfaction at adjacent time points (T1 and T2, T2 and T3) and between depressive symptoms at T3 and depressive symptoms at T1 and T2, separately. Body dissatisfaction at T3 was positively correlated with depressive symptoms at T1 and T3, separately. Body dissatisfaction and depressive symptoms were correlated at T2. Three covariates had significant or marginally significant correlations with body dissatisfaction or depressive symptoms: sex, age and intervention. They were included in the CLPM.

Similar to the overweight/obese group, we conducted a cross-lagged model with both autoregressive path and cross-lagged paths freely estimated with covariates. The regression coefficients were deleted from the model with ps>0.10 between the covariates and the outcomes, leaving the regression of body dissatisfaction at T1 on sex, body dissatisfaction at T3 on intervention and depressive symptoms at T1 on age. The T1-T2 time interval was shorter than T2-T3 time interval (mean 0.85 vs. 1.13 years, p<0.001). Therefore, we did not test the equality in the cross-lagged paths. The model fit the data well (Table 3).

In the CPLM, there was no relation between antecedent depressive symptoms and later body dissatisfaction or antecedent body dissatisfaction and later depressive symptoms (Table 3). Body dissatisfaction and depressive symptoms showed moderate stability over time (β = 0.43–0.48 for body dissatisfaction and β = 0.52–0.63 for depressive symptoms, Table 3).

#### The moderating effect of sex among healthy weight group

Body dissatisfaction significantly differed by sex at T1 (mean -0.80 females vs. -0.12 males, p = 0.034). However, on average, the sex subgroups in the healthy weight group did not have strong desires to change their body weight. No significant sex differences were found for body dissatisfaction at T2 or T3, or depressive symptoms at T1-T3. The moderating effect of sex on the relations was assessed by multiple-group CLPM. No significant sex differences were found for either the cross-lagged paths (Wald test X^2^ = 2.221, df = 4, p = 0.695) or the autoregressive paths (Wald test X^2^ = 1.739, df = 4, p = 0.784).

## Discussion

This study has two main findings among Black adolescents from low-income communities. First, body dissatisfaction differed by weight status, but depressive symptoms did not. Second, there were bidirectional relations between body dissatisfaction and depressive symptoms in the overweight/group, not in the healthy weight group. The exploratory analyses found no sex variation in the relations between body dissatisfaction and depressive symptoms, regardless of body weight.

First, Black adolescents in the overweight/obese group had high body dissatisfaction compared to Black adolescents of healthy weight. There is evidence suggesting that Black adolescents are less vulnerable to the pressures related to thinness ideal, compared to White adolescents [52]. In addition, over three fourth of the mothers were overweight or obese in the sample of Black adolescents from low-income families. Thus, one might expect that the Black adolescents from low-income families might be protected from the thinness ideal. However, this study found that Black adolescents of overweight/obese status wished to reduce their weight by 2 silhouettes on average. In addition, adolescents of overweight/obese status had significantly higher body dissatisfaction than adolescents of healthy weight. This finding is consistent with a report that increased weight was associated with greater body dissatisfaction among Black preadolescent girls [53]. The higher body dissatisfaction among the overweight/obese group suggests that the COBWEBS model applies to Black adolescents. Black adolescents were not immune from the weight-related psychosocial impact [54]. Although Black adolescents might have fewer concerns with body size than White adolescents[55], this study is consistent with other studies reporting that Black adolescents also view their overweight/obese status as a stigma [53].

In contrast, depressive symptoms did not differ significantly by weight status and depressive symptoms decreased over time for both groups. Although the healthy weight group was in general satisfied with their body weight, other psychosocial, or environmental factors might induce stress and lead to depressive symptoms [56] in the healthy weight group, given that early adolescence occurs around the onset of puberty and is marked by dramatic changes in hormone level. The decrease in depressive symptoms among the adolescents may reflect increasing social maturity with age and the ability to handle psychosocial stressors.

Second, there were bidirectional relations between body dissatisfaction and depressive symptoms among overweight/obese adolescents. The positive relation between antecedent body dissatisfaction and later depressive symptoms is consistent with a report that Black adolescents with high body dissatisfaction have high depressive symptoms [57]. However, it is contradictory to a cross-sectional study that reported a lack of relationship between body dissatisfaction and depressive symptoms among Black adolescents mixed of healthy weight and overweight/obese status [35]. The positive relation between antecedent body dissatisfaction and later depressive symptoms supports the “thinness ideal theory” that overweight/obesity increases body dissatisfaction, and the weight-related concern or pressure [21, 58] in turn leads to depressive symptoms[25, 27]. Future studies need to assess whether the relation holds among Black adolescents from middle-class families who may have mothers with a lower prevalence of overweight/obesity.

In addition, this study found that antecedent depressive symptoms predicted later body dissatisfaction in adolescents with overweight/obesity. This finding is consistent with a review article on cognition and depression [29] and with several other prospective studies on mainly White adolescents at different stages of development [9, 32]. Findings support the theory that negative self-appraisals are associated with depressive symptoms. Depressive symptoms involve a negative view of life [59] and can lead to body dissatisfaction if they are reflected on body weight. Since body dissatisfaction is a risk factor of disordered eating among Black adolescents, the finding that depressive symptoms predicted body dissatisfaction in Black adolescents with overweight/obesity suggests that prevention and treatment of eating disorders among Black adolescents should consider the prevention of depressive symptoms in addition to the reduction of body dissatisfaction.

Third, there were no relations between body dissatisfaction and depressive symptoms among the adolescents of healthy weight. Among this sample of Black adolescents in early adolescence, three-quarters of the adolescents of healthy weight were satisfied with their body size while 60% of overweight/obese adolescents were not satisfied with their body size. Low body dissatisfaction may explain the lack of the positive association with antecedent body dissatisfaction and later depressive symptoms. Interiorization in Black adolescents of a corporal aesthetic may differ from that of Whites in the healthy weight group [60]. In White adolescents, especially females, body dissatisfaction has a normative presence. One study found that one third of non-Hispanic White girls were dissatisfied with their body weight, even when their BMI was at or below the age- and sex-specific median, while Black adolescents of healthy weight were more likely to accept their body weight [60].

Finally, this study explored the moderating effect of sex on the relations between body dissatisfaction and depressive symptoms. No sex variation was found for either weight group. Previous studies assessed the variation in the relation between body dissatisfaction and depressive symptoms by weight status or sex, but not both. The lack of significant differences in body dissatisfaction or depressive symptoms by sex in Black early adolescents is consistent with a study on body dissatisfaction among Black adolescents of ~13 years [61], but contradictory to a study reporting a higher prevalence of depressive symptoms among females than males in a national representative sample of adolescents aged 12–17 years [62]. The finding that there was no significant moderating effect of sex for either weight group is contradictory to the two studies that found sex variation in the bidirectional relations between body dissatisfaction and depressive symptoms [30, 31]. The high body dissatisfaction among adolescent males and the lack of variation by sex for either weight group suggest that prevention of psychological disorders among Black adolescents with overweight/obesity should not ignore the psychosocial impact for adolescent males.

### Strengths

This study has several strengths. First, it is a prospective study that examined the bidirectional relations between body dissatisfaction and depressive symptoms in Black early adolescents from low-income families. It assessed the relations for adolescents with overweight/obesity or adolescents of healthy weight, separately. Second, a novel statistical model of CLPM was used to assess the bidirectional relations. This method allows the test of the bidirectional relations, after accounting for the stability of the same outcomes. Third, we assessed Black adolescents in early adolescence (12–13 years) from low-income families. An estimated 58% of the adolescents were under the federal poverty threshold and 78% of the mothers were overweight/obese, suggesting that the adolescents were at high risk of overweight/obesity and mental health problems. Fourth, depressive symptoms and body dissatisfaction were measured through computerized ACASI, which has been shown to increase the reporting of sensitive behaviors [63].

### Limitations

This study has several limitations. First, the modest sample size for weight status limits the power to assess sex differences in the relations. Future studies need to recruit larger samples to assess the sex differences. Second, half of the families lived below the federal poverty threshold, increasing their risk for both depressive symptoms [64], and overweight/obesity[3]. The findings should be replicated before being generalized to Black adolescents from other socio-demographic backgrounds, e.g. adolescent of middle-class families. Third, although the BDI has been conducted among adolescents, we recommend considering the CDI, which was developed for children, for future studies [65]. Fourth, the temporal relation between body dissatisfaction and depressive symptoms in either direction does not necessarily indicate a causal relation.

### Conclusion and implications

In summary, the reciprocal body dissatisfaction–depressive symptom relations among Black adolescents with overweight/obesity, but not their counterparts of healthy weight, suggest that overweight/obesity is a stressor in development of serious psychological disorders, e.g. body dissatisfaction and depressive symptoms among Black early adolescents, similar to adolescents of other racial/ethnic groups.

The findings have implications for mental health and obesity prevention intervention programs among Black adolescents. First, the high rates of overweight/obesity among Black adolescents and the elevated mental health problems among the overweight/obese group highlight the need to prevent overweight/obesity among Black early adolescents. Overweight/obesity prevention programs may encourage the black adolescents to recognize personal strengths other than body weight[66, 67]. Second, the bidirectional relations between body dissatisfaction and depressive symptoms among the overweight/obese group suggest that prevention of body dissatisfaction and depressive symptoms may be mutually beneficial and potentially combined. Treatment and prevention of depressive symptoms may prevent the onset of body dissatisfaction and disordered eating, and prevention of body dissatisfaction might prevent the development of depressive symptoms among Black adolescents with overweight/obesity. Third, a lack of sex differences in the relations suggests that prevention of mental health disorder in Black early adolescents should include both sexes.

## Supporting information

S1 File Protocol of this research. (DOCX)

## References

[1] Skinner AC, Perrin EM, Skelton JA. Prevalence of obesity and severe obesity in US children, 1999–2014. Obesity (Silver Spring, Md). 2016;24(5):1116–23. Epub 2016/04/27. 10.1002/oby.21497 . 27112068

[2] Rogers R, Eagle TF, Sheetz A, Woodward A, Leibowitz R, Song M, et al The Relationship between Childhood Obesity, Low Socioeconomic Status, and Race/Ethnicity: Lessons from Massachusetts. Childhood obesity (Print). 2015;11(6):691–5. Epub 2015/11/13. 10.1089/chi.2015.0029 26562758 PMC4939441

[3] U.S. Department of Health and Human Services Health Resources and Services Administration; Maternal and Child Health Bureau. Child Health USA 2014: Adolescent Overweight and Obesity: Data. Rockville, Maryland: 2015.

[4] Tomiyama AJ. Weight stigma is stressful. A review of evidence for the Cyclic Obesity/Weight-Based Stigma model. Appetite. 2014;82:8–15. 10.1016/j.appet.2014.06.108 24997407

[5] Smolak L, Thompson K. Body image, eating disorders, and obesity in youth. assessment, prevention, and treatment (2nd ed.). Washington, DC: American Psychological Association 2009.

[6] Stice E, Presnell K, Spangler D. Risk factors for binge eating onset in adolescent girls: a 2-year prospective investigation. Health psychology: official journal of the Division of Health Psychology, American Psychological Association. 2002;21(2):131–8. Epub 2002/04/13. . 11950103

[7] Glied S, Pine DS. Consequences and Correlates of Adolescent Depression. Archives of Pediatrics & Adolescent Medicine. 2002;156(10):1009–14. 10.1001/archpedi.156.10.1009 12361447

[8] Johnson D, Dupuis G, Piche J, Clayborne Z, Colman I. Adult mental health outcomes of adolescent depression: A systematic review. Depression and anxiety. 2018;35(8):700–16. Epub 2018/06/08. 10.1002/da.22777 . 29878410

[9] Bearman SK, Martinez E, Stice E, Presnell K. The Skinny on Body Dissatisfaction: A Longitudinal Study of Adolescent Girls and Boys. Journal of youth and adolescence. 2006;35(2):217–29. Epub 2006/08/17. 10.1007/s10964-005-9010-9 16912810 PMC1540456

[10] Center for Behavioral Health Statistics and Quality. 2017 National Survey on Drug Use and Health Final Analytic File Codebook, Substance Abuse and Mental Health Services Administration, Rockville, MD. 2018.

[11] Stice E, Whitenton K. Risk factors for body dissatisfaction in adolescent girls: A longitudinal investigation. Developmental Psychology. 2002;38(5):669–78. 10.1037//0012-1649.38.5.669 12220046

[12] Quek YH, Tam WW, Zhang MW, Ho RC. Exploring the association between childhood and adolescent obesity and depression: a meta‐analysis. Obesity reviews. 2017;18(7):742–54. 10.1111/obr.12535 28401646

[13] Fung SS, Stewart SM, Ho SY, Wong JP, Lam TH. Body dissatisfaction, maternal appraisal, and depressive symptoms in Hong Kong adolescents. International journal of psychology: Journal international de psychologie. 2010;45(6):453–60. Epub 2011/11/03. 10.1080/00207594.2010.481719 . 22044085

[14] Duchesne AP, Dion J, Lalande D, Begin C, Emond C, Lalande G, et al Body dissatisfaction and psychological distress in adolescents: Is self-esteem a mediator? Journal of health psychology. 2017;22(12):1563–9. Epub 2016/03/02. 10.1177/1359105316631196 . 26929171

[15] Flores-Cornejo F, Kamego-Tome M, Zapata-Pachas MA, Alvarado GF. Association between body image dissatisfaction and depressive symptoms in adolescents. Revista brasileira de psiquiatria (Sao Paulo, Brazil: 1999). 2017;39(4):316–22. Epub 2017/03/30. 10.1590/1516-4446-2016-1947 . 28355343 PMC7111410

[16] Solomon-Krakus S, Sabiston CM, Brunet J, Castonguay AL, Maximova K, Henderson M. Body Image Self-Discrepancy and Depressive Symptoms Among Early Adolescents. Journal of Adolescent Health. 2017;60(1):38–43. 10.1016/j.jadohealth.2016.08.024 27793726

[17] Thompson JK, Stice E. Thin-Ideal Internalization: Mounting Evidence for a New Risk Factor for Body-Image Disturbance and Eating Pathology. Current Directions in Psychological Science. 2001;10(5):181–3. 10.1111/1467-8721.00144

[18] Morrison TG, Kalin R, Morrison MA. Body-image evaluation and body-image investment among adolescents: a test of sociocultural and social comparison theories. Adolescence. 2004;39(155):571–92. Epub 2005/01/28. . 15673231

[19] Mitchell SH, Petrie TA, Greenleaf CA, Martin SB. Moderators of the internalization–body dissatisfaction relationship in middle school girls. Body Image. 2012;9(4):431–40. 10.1016/j.bodyim.2012.07.001 22858554

[20] Jeffers AJ, Cotter EW, Snipes DJ, Benotsch EG. BMI and depressive symptoms: The role of media pressures. Eating Behaviors. 2013;14(4):468–71. 10.1016/j.eatbeh.2013.08.007 24183138

[21] Knauss C, Paxton SJ, Alsaker FD. Relationships amongst body dissatisfaction, internalisation of the media body ideal and perceived pressure from media in adolescent girls and boys. Body Image. 2007;4(4):353–60. Epub 2007/12/20. 10.1016/j.bodyim.2007.06.007 . 18089281

[22] Ohring R, Graber JA, Brooks-Gunn J. Girls' recurrent and concurrent body dissatisfaction: correlates and consequences over 8 years. The International journal of eating disorders. 2002;31(4):404–15. Epub 2002/04/12. 10.1002/eat.10049 . 11948645

[23] Ferreiro F, Seoane G, Senra C. A prospective study of risk factors for the development of depression and disordered eating in adolescents. Journal of clinical child and adolescent psychology: the official journal for the Society of Clinical Child and Adolescent Psychology, American Psychological Association, Division 53. 2011;40(3):500–5. Epub 2011/05/03. 10.1080/15374416.2011.563465 . 21534061

[24] Ferreiro F, Seoane G, Senra C. Gender-related risk and protective factors for depressive symptoms and disordered eating in adolescence: a 4-year longitudinal study. Journal of youth and adolescence. 2012;41(5):607–22. Epub 2011/10/04. 10.1007/s10964-011-9718-7 . 21965131

[25] Stice E, Bearman SK. Body-image and eating disturbances prospectively predict increases in depressive symptoms in adolescent girls: a growth curve analysis. Dev Psychol. 2001;37(5):597–607. Epub 2001/09/13. 10.1037//0012-1649.37.5.597 . 11552756

[26] Paxton SJ, Neumark-Sztainer D, Hannan PJ, Eisenberg ME. Body dissatisfaction prospectively predicts depressive mood and low self-esteem in adolescent girls and boys. Journal of clinical child and adolescent psychology: the official journal for the Society of Clinical Child and Adolescent Psychology, American Psychological Association, Division 53. 2006;35(4):539–49. Epub 2006/09/30. 10.1207/s15374424jccp3504_5 . 17007599

[27] Sharpe H, Griffiths S, Choo TH, Eisenberg ME, Mitchison D, Wall M, et al The relative importance of dissatisfaction, overvaluation and preoccupation with weight and shape for predicting onset of disordered eating behaviors and depressive symptoms over 15 years. The International journal of eating disorders. 2018;51(10):1168–75. Epub 2018/09/09. 10.1002/eat.22936 30194690 PMC6289784

[28] Prieto SL, Cole DA, Tageson CW. Depressive self-schemas in clinic and nonclinic children. Cognitive Therapy and Research. 1992;16(5):521–34. 10.1007/bf01175139

[29] Gotlib IH, Joormann J. Cognition and depression: current status and future directions. Annual review of clinical psychology. 2010;6:285–312. Epub 2010/03/03. 10.1146/annurev.clinpsy.121208.131305 20192795 PMC2845726

[30] Patalay P, Sharpe H, Wolpert M. Internalising symptoms and body dissatisfaction: untangling temporal precedence using cross-lagged models in two cohorts. Journal of child psychology and psychiatry, and allied disciplines. 2015;56(11):1223–30. Epub 2015/04/24. 10.1111/jcpp.12415 . 25902846

[31] Sharpe H, Patalay P, Choo TH, Wall M, Mason SM, Goldschmidt AB, et al Bidirectional associations between body dissatisfaction and depressive symptoms from adolescence through early adulthood. Development and psychopathology. 2018;30(4):1447–58. Epub 2017/11/17. 10.1017/S0954579417001663 29144209 PMC6343674

[32] Paxton SJ, Eisenberg ME, Neumark-Sztainer D. Prospective predictors of body dissatisfaction in adolescent girls and boys: a five-year longitudinal study. Dev Psychol. 2006;42(5):888–99. Epub 2006/09/07. 10.1037/0012-1649.42.5.888 . 16953694

[33] Franko DL, Striegel-Moore RH. The role of body dissatisfaction as a risk factor for depression in adolescent girls: are the differences Black and White? Journal of psychosomatic research. 2002;53(5):975–83. Epub 2002/11/26. 10.1016/s0022-3999(02)00490-7 . 12445587

[34] Kelly NR, Bulik CM, Mazzeo SE. An exploration of body dissatisfaction and perceptions of Black and White girls enrolled in an intervention for overweight children. Body Image. 2011;8(4):379–84. Epub 2011/06/28. 10.1016/j.bodyim.2011.05.003 21700518 PMC3170454

[35] Young-Hyman D, Tanofsky-Kraff M, Yanovski SZ, Keil M, Cohen ML, Peyrot M, et al Psychological status and weight-related distress in overweight or at-risk-for-overweight children. Obesity (Silver Spring, Md). 2006;14(12):2249–58. Epub 2006/12/26. 10.1038/oby.2006.264 17189553 PMC1862955

[36] Almeida S, Severo M, Araujo J, Lopes C, Ramos E. Body image and depressive symptoms in 13-year-old adolescents. Journal of paediatrics and child health. 2012;48(10):E165–71. Epub 2012/09/25. 10.1111/j.1440-1754.2012.02576.x . 22998142

[37] Chen G, Guo G, Gong J, Xiao S. The Association Between Body Dissatisfaction and Depression: An Examination of the Moderating Effects of Gender, Age, and Weight Status in a Sample of Chinese Adolescents. Journal of Psychologists and Counsellors in Schools. 2015;25(2):245–60. Epub 04/27. 10.1017/jgc.2015.6

[38] McCreary DR, Sasse DK. An exploration of the drive for muscularity in adolescent boys and girls. Journal of American college health: J of ACH. 2000;48(6):297–304. Epub 2000/06/23. 10.1080/07448480009596271 . 10863873

[39] Black MM, Hager ER, Le K, Anliker J, Arteaga SS, DiClemente C, et al Challenge! Health Promotion/Obesity Prevention Mentorship Model Among Urban, Black Adolescents. Pediatrics. 2010;126(2):280–8. 10.1542/peds.2009-1832 20660556 PMC4124131

[40] Kuczmarski RJ, Ogden CL, Guo SS, Grummer-Strawn LM, Flegal KM, Mei Z, et al 2000 CDC Growth Charts for the United States: methods and development. Vital and health statistics Series 11, Data from the National Health Survey. 2002;(246):1–190. Epub 2002/06/05. . 12043359

[41] Beck AT, Ward CH, Mendelson M, Mock J, Erbaugh J. An inventory for measuring depression. Archives of General Psychiatry. 1961;4:561–71. 10.1001/archpsyc.1961.01710120031004 13688369

[42] Bennett DS, Ambrosini PJ, Bianchi M, Barnett D, Metz C, Rabinovich H. Relationship of Beck Depression Inventory factors to depression among adolescents. Journal of affective disorders. 1997;45(3):127–34. Epub 1997/09/23. 10.1016/s0165-0327(97)00045-1 . 9298425

[43] Stunkard AJ, Sørensen T, Schulsinger F. Use of the Danish Adoption Register for the study of obesity and thinness. Research publications—Association for Research in Nervous and Mental Disease. 1983;60:115–20. . Language: English. Date Revised: 20041117. Date Created: 19830317. Date Completed: 19830317. Update Code: 20121129. Publication Type: Journal Article. Journal ID: 7505942. Publication Model: Print. Cited Medium: Print. NLM ISO Abbr: Res Publ Assoc Res Nerv Ment Dis. Linking ISSN: 00917443. Subset: IM. 6823524

[44] Mitola AL, Papas MA, Le K, Fusillo L, Black MM. Agreement with satisfaction in adolescent body size between female caregivers and teens from a low-income African-American community. Journal of pediatric psychology. 2007;32(1):42–51. 10.1093/jpepsy/jsl004 . Language: English. Date Revised: 20071203. Date Created: 20061228. Date Completed: 20070301. Update Code: 20121129. Publication Type: Journal Article. Journal ID: 7801773. Publication Model: Print-Electronic. Cited Medium: Print. NLM ISO Abbr: J Pediatr Psychol. Linking ISSN: 01468693. Subset: IM. 16762992

[45] Bureau USC. Poverty Thresholds 2004. 2012.

[46] Cuzick J. A Wilcoxon-type test for trend. Statistics in Medicine. 1985; 4: 87–90. 10.1002/sim.4780040112 3992076

[47] Conover WJ. Practical Nonparametric Statistics, 3rd ed New York: Wiley 1999.

[48] Kenny DA. Cross-lagged panel correlation: A test for spuriousness. Psychological Bulletin. 1975;82(6):887–903. 10.1037/0033-2909.82.6.887

[49] Muthén LKaM, B.O. Mplus User’s Guide. Eighth Edition Los Angeles, CA: Muthén & Muthén1998-2017.

[50] Kline RB. Principles and Practice of Structural Equation Modeling 2005.

[51] Little T. Longitudinal structural equation modeling. New York: Guilford Press; 2013.

[52] Nichter M. Fat Talk: What Girls and their Parents Say about Dieting. Cambridge, MA: Harvard University Press 2000.

[53] Tyler C, A. Johnston C, Dalton W, Foreyt J. Relationships Between Weight and Body Dissatisfaction, Body Esteem, and Teasing in African American Girls 2009. 125–32 p.

[54] Granberg EM, Simons RL, Gibbons FX, Melby JN. The Relationship between Body Size and Depressed Mood: Findings from a Sample of African American Middle School Girls. Youth & society. 2008;39(3):294–315. Epub 2008/03/01. 10.1177/0044118X07301952 19834569 PMC2761634

[55] Beauboeuf-Lafontant T. Strong and large Black women? Exploring relationships between deviant womanhood and weight. Gender & Society. 2003;17(1):111–21.

[56] Thapar A, Collishaw S, Pine DS, Thapar AK. Depression in adolescence. Lancet. 2012;379(9820):1056–67. Epub 02/02. 10.1016/S0140-6736(11)60871-4 . 22305766 PMC3488279

[57] Bucchianeri MM, Fernandes N, Loth K, Hannan PJ, Eisenberg ME, Neumark-Sztainer D. Body dissatisfaction: Do associations with disordered eating and psychological well-being differ across race/ethnicity in adolescent girls and boys? Cultural Diversity and Ethnic Minority Psychology. 2016;22(1):137–46. 10.1037/cdp0000036 26052976 PMC6712990

[58] Petrie TA, Greenleaf C, Martin S. Biopsychosocial and physical correlates of middle school boys’ and girls’ body satisfaction. Sex Roles: A Journal of Research. 2010;63(9–10):631–44. 10.1007/s11199-010-9872-5

[59] Beck AT, Ward CH, Mendelson M, Mock J, Erbaugh J. An inventory for measuring depression. Arch Gen Psychiatry. 1961;4:561–71. Epub 1961/06/01. 10.1001/archpsyc.1961.01710120031004 . 13688369

[60] Mikolajczyk RT, Iannotti RJ, Farhat T, Thomas V. Ethnic differences in perceptions of body satisfaction and body appearance among U.S. schoolchildren: a cross-sectional study. BMC public health. 2012;12:425 Epub 2012/06/14. 10.1186/1471-2458-12-425 22691404 PMC3490835

[61] Jones LR, Fries E, Danish SJ. Gender and ethnic differences in body image and opposite sex figure preferences of rural adolescents. Body Image. 2007;4(1):103–8. Epub 2007/12/20. 10.1016/j.bodyim.2006.11.005 18089257 PMC2031852

[62] Ghandour RM, Sherman LJ, Vladutiu CJ, Ali MM, Lynch SE, Bitsko RH, et al Prevalence and Treatment of Depression, Anxiety, and Conduct Problems in US Children. The Journal of Pediatrics. 2019;206:256–67.e3. 10.1016/j.jpeds.2018.09.021 30322701 PMC6673640

[63] Moum T. Mode of administration and interviewer effects in self-reported symptoms of anxiety and depression. Social Indicators Research. 1998;(45):279–318.

[64] Pratt LA, Brody DJ. Depression in the U.S. household population, 2009–2012. NCHS data brief. 2014;(172):1–8. Epub 2014/12/04. . 25470183

[65] Kovacs M. Children's Depression Inventory. North Tonawanda, NY: Multi-Health Systems, Inc.; 1992.

[66] Pesa JA, Syre TR, Jones E. Psychosocial differences associated with body weight among female adolescents: the importance of body image. The Journal Of Adolescent Health. 2000;26(5):330–7. 10.1016/s1054-139x(99)00118-4 . Language: English. Date Revised: 20061115. Date Created: 20000524. Date Completed: 20000524. Update Code: 20121129. Publication Type: Comparative Study. Journal ID: 9102136. Publication Model: Print. Cited Medium: Print. NLM ISO Abbr: J Adolesc Health. Linking ISSN: 1054139X. Subset: IM. 10775825

[67] Neumark-Sztainer D, Levine MP, Paxton SJ, Smolak L, Piran N, Wertheim EH. Prevention of body dissatisfaction and disordered eating: What next? Eating Disorders. 2006;14(4):265–85. 10.1080/10640260600796184 . Language: English. Date Created: 20060728. Date Completed: 20061222. Update Code: 20121129. Publication Type: Journal Article. Journal ID: 9315161. Publication Model: Print. Cited Medium: Print. NLM ISO Abbr: Eat Disord. Linking ISSN: 10640266. Subset: IM. 16873144

