# Supplementary material for: Longitudinal bidirectional relations between body dissatisfaction and depressive symptoms among Black adolescents: A cross-lagged panel analysis
Source: PLoS One. 2020 Jan 30;15(1):e0228585. doi: 10.1371/journal.pone.0228585 (PMC6992219; doi:10.1371/journal.pone.0228585)
Supplement: S1 File — (DOCX) [file pone.0228585.s001.docx]

**PROTOCOL**

**1. Purpose**

1) To compare body dissatisfaction and depressive symptoms by weight status (overweight/obesity vs. healthy weight) among Black adolescents 12-13 years old.

2) To investigate the relationship between body dissatisfaction and depressive symptoms among Black adolescents 12-13 years old, who were overweight or obese.

3) To investigate the relationship between body dissatisfaction and depressive symptoms among Black adolescents 12-13 years old, who were of healthy weight.

***Target variables:***

- Body dissatisfaction: discrepancy between perceived and ideal body weight and shape
- Depressive symptoms

**2. Inclusion Criteria**

1. A sample of adolescents in middle schools, who were recruited to participate in a random controlled trial on overweight/obesity prevention, promoting healthy diet and physical activity.
2. The adolescents who were Black.
3. The adolescents who were 12-13 years old at the first assessment (baseline)
4. The adolescents who were healthy weight (5-85^th^ BMI percentile) or overweight/obese (>=85^th^ BMI percentile).
5. Both sexes.

**3. Exclusion Criteria**

1) The adolescents who were not Black

2) The adolescents who were younger than 12 years or older than 13 years at baseline

3) The adolescents who were underweight (<=5th BMI percentile)

**4. Sample Size and Enrollment Period**

　Sample size: 153 adolescents (overweight/obesity 57, healthy weight: 96)

　Enrollment period: 7/1/2002-4/16/2004. Then they were assessed again about 10 and 24 months later.

**5. Study Design**

Longitudinal with three assessments: baseline (T1, 7/1/2002-4/16/2004), ~10 months (T2, 12/2/2002-11/4/2005) and ~24 months after baseline (T3, 2/3/2004-11/7/2006)

**6. Study Participation and Patient Registration / Allocation Procedures**

(1) The obesity prevention intervention trial has been registered with the Clinical Trials Registry. (ClinicalTrials.gov Identifier: NCT00746083).

(2) The University of Maryland Baltimore Institutional Review Board approved this research;

(3) All parents signed informed consent, and all adolescents signed informed assent.

**7. Surveys / Questionnaires**

Data on socio-demographic characteristics, body dissatisfaction and depressive symptoms were collected through computerized audio computer-assisted self-interview (ACASI).

**8. Measures**

1. Adolescent characteristics

Sex, age, race/ethnicity, height, body weight, birthdate, poverty, maternal education, maternal overweight/obesity and intervention status.

1. Depressive symptoms

- Depressive symptoms were measured with the Beck Depression Inventory (BDI)
- Consisting of 21 items relating to depressive symptoms in the past week, e.g., sadness, feeling guilty, and fatigue, using a 4-point response scale of severity.
- A summary score was calculated with higher scores indicating more depressive symptoms.

1. Body dissatisfaction

- Body dissatisfaction was measured by comparing perceived body image with ideal image, using a culturally adapted, age- and sex-specific, 9-point silhouette scale, modified from Stunkard, Sørensen, & Schulsinger, (1983) to more closely resemble Black youth.
- The silhouettes were ordered from thinnest (rating=1) to heaviest (rating=9). The adolescents identified the silhouette closest to their current body size and then on a separate, but identical scale, the silhouette they desired.
- The discrepancy score was defined as perceived body size minus desired body size, with a positive discrepancy score indicating desire for a thinner body and a negative score indicating desire to be heavier.

**9. Analysis**

- T-test for continuous variable and Chi-square tests (or Fisher’s exact test) for categorical variable by weight status

Analysis

Data entry

- Pearson correlation coefficients among body dissatisfaction and depressive symptoms over time and the covariates.
- Trend test for change of body dissatisfaction and depressive symptoms over time
- Cross-lagged panel model (CLPM) to examine the reciprocal relations between body dissatisfaction and depressive symptoms, accounting for the stability of depressive symptoms or body dissatisfaction across time.
